# Bilingualism Effects on the Cognitive Flexibility of Autistic Children: Evidence From Verbal Dual-Task Paradigms

**DOI:** 10.1162/nol_a_00055

**Published:** 2021-12-23

**Authors:** Eleni Peristeri, Margreet Vogelzang, Ianthi Maria Tsimpli

**Affiliations:** Department of Neurology, University Hospital of Larissa, Faculty of Medicine, University of Thessaly, Larissa, Greece; Faculty of Modern and Medieval Languages and Linguistics, University of Cambridge, Cambridge, UK

**Keywords:** autism, bilingualism, cognitive flexibility, dual-task, executive functions, language ability

## Abstract

The deficit in cognitive flexibility (i.e., the ability to adapt cognitive behavior to changing contexts) is one of the most prominent characteristics of autistic individuals. Inflexibility may manifest in restricted interests and increased susceptibility to the effects of misinformation either through inefficient inhibition of non-target information or deficient recall of correct information. Bilingualism has been shown to enhance executive functions in both typically developing children and autistic children; yet, the effect of bilingualism on cognitive flexibility in autism remains underexplored. In this study, we used verbal dual-tasks to compare cognitive flexibility across 50 monolingual autistic and 50 bilingual autistic children, and 50 monolingual and 50 bilingual typically developing children. The children were also administered language ability tests and a nonverbal global-local cognitive flexibility task, in order to investigate whether performance in the dual-tasks would be modulated by the children’s language and executive function skills. The bilingual autistic children outperformed their monolingual autistic peers in the dual-tasks. The strength of the bilingualism effect, however, was modulated by the type of language processing that interfered with the target information in each dual-task, which suggests that the bilingual autistic children calibrated their processing resources and efficiently adapted them to the changing demands of the dual-task only to the extent that the task did not exceed their language abilities. Bilingual autistic children relied on their executive functions rather than on their language abilities while performing in the dual-tasks. The overall results show that bilingualism compensates for the reduced cognitive flexibility in autism.

## INTRODUCTION

Prior research shows that autism has an adverse effect on cognition, including cognitive flexibility skills (de Vries & Geurts, 2012; Van Eylen et al., 2011). This impairment has been attributed to executive function (EF) deficits in autism that make performance under conditions of divided attention cognitively demanding (Garcia-Villamistar & Sala, 2002; Goldberg et al., 2005; Landa & Goldberg, 2005; see Geurts et al., 2014 for a review). On the other hand, recent research (Gonzalez-Barrero & Nadig, 2019; Iarocci et al., 2017; Peristeri et al., 2020; Sharaan et al., 2020) has shown that bilingualism has a positive effect on a number of EFs in autism, including inhibition, flexible switching, and sustained attention, though other research found no such advantage (Li et al., 2017). In the current study, we examined the impact of bilingualism on the performance of autistic children and typically developing (TD) children in three language tasks that measure flexibility, since in all of them, participants were expected to show flexible behaviors. More specifically, across the three tasks, children had to recall words and then switch to a new rule (i.e., semantic judgment, syntactic processing, counting aloud) within the same trials. Besides testing bilingualism effects in cognitive flexibility in autism, the distinct language demands of the three tasks allowed us to investigate whether bilingualism effects in flexibility in language vary based on the type of language processing that the children had to perform when switching to the task’s new rule. Furthermore, we assessed the extent to which inter-individual variability in the children’s chronological age, autism symptomatology, socio-economic status (SES), intelligence (IQ), EF, language ability, and dual-language experience metrics (for bilingual autistic children and bilingual TD children only) is related to their cognitive flexibility performance in the three dual-tasks.

### Executive Functions in Autism

Deficits in executive functioning, an ability that encompasses inhibition and planning (Ozonoff et al., 1991; Pellicano et al., 2006), as well as attention regulation, set shifting, and working memory (Solomon et al., 2017), have been often observed in autistic children and adolescents. Previous research using experimental paradigms measuring inhibition has routinely demonstrated that autistic children exhibit more erroneous and slower responses than TD peers in incongruent (vs. congruent) trials, in which the relevant and irrelevant stimulus dimensions do not prompt the same response (Kornblum & Lee, 1995). In fact, response errors and/or increases in response latencies in the face of manipulations of incompatible stimulus activation have been taken to reflect extra effort needed to inhibit interference from competing stimuli; during EF tasks, the processing of the irrelevant, distracting stimulus interferes with the target stimulus and has to be inhibited for a relatively short time interval. It is assumed that this requires higher-order executive processes accompanied by increased cognitive effort (e.g., Posner & Cohen, 1984; Tipper et al., 1994). Interference control impairments have been typically found in autistic children (Bryson, 1983; Christ et al., 2007; Geurts et al., 2014; Sanderson & Allen, 2013), which, in principle, means that task-unrelated stimuli interfere with task-relevant information more strongly in autism relative to neurotypical controls. Importantly, such difficulties have so far been mainly shown in the non-linguistic, cognitive domain. For instance, autistic children in Peristeri et al.’s (2020) study exhibited higher rates of false hits and slower response times than age-matched TD children in a 2-back task with digits, in which incoming digits that did not match the digit in trial n–2 had to be inhibited. Also, Adams and Jarrold’s (2012) study showed that autistic children had poorer performance in the conflict trials of a flanker task comparable to TD and cognitively disabled children used as the control groups.

Individual differences in emerging EF within the autistic population have provided a means of understanding heterogeneity in early symptom expression. Poor inhibitory control has been particularly implicated in the maintenance of inappropriate (i.e., non-adaptive) thoughts and behaviors distinctive of autism, which has been suggested to underpin core features of restrictive and repetitive behaviors, such as insistence on sameness (Turner, 1997, 1999). Where studies have managed to run tests of EF that yield substantial variance, this tends to be associated with individual differences in age and IQ, especially for inhibition, interference control, and set-shifting tasks. For example, in Geurts et al.’s (2014) meta-analysis, chronological age and IQ were found to moderate autistic individuals’ performance on prepotent response inhibition and interference control, respectively, while other research (Memari et al., 2013) has shown that set-shifting skills were rather affected by the children’s gender and intellectual ability, but not age. High-functioning autistic children in Van Eylen and colleagues’ (2011) study experienced greater switch costs in set shifting relative to the neurotypical group; yet, the authors acknowledge that this effect was difficult to measure reliably due to the large heterogeneity in the autistic group in terms of both demographic (wide age range: 8 to 18 years old) and clinical characteristics (variance in IQ scores). Heterogeneity in EF in autism has been verified by Leung and Zakzanis’ (2014) meta-analysis, which has exhibited considerable variability of effect size in set-shifting performance across autistic populations, further implying that EF deficits do not constitute a uniform characteristic of the autism spectrum. Besides heterogeneous demographic, clinical, and behavioral characteristics, which potentially affect the findings on EF in autism, another challenge to overcome is that the EF measures of interest rarely stand in isolation from one another, since performance on specific EF components often call on related sub-processes (Van Eylen et al., 2011). A mixture of overlapping EF sub-processes across experimental paradigms makes it rather difficult to disentangle heterogeneity in EF in autism.

### Cognitive Flexibility in Autism

Cognitive flexibility is a common feature of EF and refers to the ability to adapt cognitive behavior in response to changing context demands in order to maximize successful performance in a particular cognitive task or situation (Deák & Wiseheart, 2015; Ionescu, 2012). Though various EF skills are often nested in cognitive flexibility, shifting has been treated as being synonymous with this cognitive skill, as both necessitate rapid transitions from one task to another, and a reconfiguration of processing mode and task sets in response to the changing context (Vandierendonck et al., 2010). Dual-task paradigms, in which two different tasks have to be performed concurrently or sequentially, have been used to quantify cognitive flexibility (among other tasks), since these paradigms tap into the individual’s ability to simultaneously consider multiple conflicting representations in two tasks. The main measure of cognitive flexibility in dual-task paradigms is the switching cost, which stems from the need to rapidly change from one rule or task to another when giving a response, and, thus, resolve interference from the previous task set (Koch et al., 2005). However, the fact that various EF skills are often nested in cognitive flexibility, combined with the high predictability in the switching measures used (de Vries & Geurts, 2012; Van Eylen et al., 2011), makes it difficult to identify the true effect size of cognitive flexibility deficits in autism. So far, the current literature has not reached a consensus on what cognitive flexibility exactly means and how to best quantify it.

Cognitive flexibility impairments in autistic individuals have often been associated with their poor performance in dual-task paradigms. Children with autism in Mostert-Kerckhoffs et al.’s (2015) study needed considerably more time to inhibit distracting stimuli in the auditory modality of an attention-shifting task. Interestingly, no interference effects emerged in the visual version of the same shifting task, which was explained in part because autistic children relied more on visual rather than verbal abilities when completing the dual-task. Also, Eigsti et al. (2008) found that autism had an adverse effect on performance in a dual-task paradigm, in which autistic participants along with TD controls were required to tap with their finger while simultaneously describing an image. Though both groups’ tapping rate was slowed-down in the dual-task as compared to the baseline single-task condition (i.e., finger tapping), the autistic group was considerably slower than controls in the dual-task condition only. Furthermore, in Remington and Fairnie’s (2017) dual-task study, autistic adults and neurotypical controls were asked to listen to a story (a conversation about a party), which was unexpectedly interrupted by the repetition of a phrase that was irrelevant to the story’s content. Though the two experimental groups did not differ in overall auditory comprehension accuracy, the percentage of the autistic participants who reported hearing the irrelevant auditory information was significantly greater than neurotypical controls. The overall evidence implies that dual-task paradigms can be particularly challenging for autistic individuals and, thus, have a robust autism-discriminative ability in research involving both children and adults. Crucially, while research in TD children has demonstrated that verbal abilities predict and mediate performance on tasks that measure cognitive flexibility both cross-sectionally (Low & Simpson, 2012) and longitudinally (Watson et al., 2001), this relationship is less understood in autism.

Though the component neural processes underlying cognitive flexibility deficits in autistic children remain unclear, atypical activation of the lateral frontoparietal and the insular network has been observed. More specifically, 7- to 14-year-old autistic children in Yerys and colleagues’ (2015) functional magnetic resonance imaging (fMRI) study demonstrated hyper-activation of frontal brain regions during a nonverbal set-shifting paradigm. This is consistent with findings of sensory hyper-responsiveness previously identified in sensory and association cortices (S. A. Green et al., 2015), suggesting that cortical hyper-excitability is also present in the frontoparietal circuit critical for cognitive flexibility. According to Yerys et al. (2015), stronger activation of the frontoparietal network in autistic (vs. TD) children suggests that it may have been harder for them to decide what to do with the feedback regarding the continuation (or not) of a behavior, thus requiring additional time to switch their responses. Importantly, these brain-based markers were not found to be linked to either the reaction times or the accuracy measure of the set-shifting task. Taylor et al.’s (2012) fMRI study has also investigated the neural underpinnings of the nonverbal set-shifting abilities of a group of 7- to 14-year-old autistic children. While age-matched TD children demonstrated typical increases in the activation of the right insula, a region which has been implicated in inhibitory control (Ghahremani et al., 2015; Swick et al., 2011), the autistic group exhibited a decrease in this activation with age. This decrease was mostly evident from 10 years of age for the autistic children. The lack of an interaction between age and right insula activation in Taylor et al.’s (2012) study demonstrates that this region is not being recruited appropriately by autistic children during inhibition. The overall results indicate significantly altered brain activation during set-shifting performance in autistic children when compared to TD children, suggesting a neurobiological difference underpinning cognitive inflexibility in autism. The current study focuses on investigating cognitive flexibility in bilingual (vs. monolingual) autistic children based on behavioral evidence; yet, future comparative research should focus on identifying potential neurophysiological markers of cognitive flexibility performance in these populations.

The consequences of cognitive flexibility impairments in autism have been mainly studied in the domain of social interactions and everyday functioning. Deficits in the ability to disengage from one task and adapt to a new one have been found to increase social anxiety, as interpersonal relations involve the rapid integration of multiple auditory and visual cues, and selecting components irrelevant to goal-directed behavior make understanding social events rather chaotic for autistic individuals (Demetriou et al., 2018; South & Rodgers, 2017). In addition, cognitive flexibility has been associated with autistic traits, since individuals with higher autism-spectrum quotient reflected in their poor social, communication, and imagination abilities, as well as in high attention to detail and effortful attention-switching (Baron-Cohen et al., 2001), were found to have poorer performance in attention-shifting tasks (Albein-Urios et al., 2018; Gökçen et al., 2014; Ridley et al., 2011). Thus, it is possible that autistic individuals with relatively low autistic traits can cope more efficiently with an adaptive or flexible behavior gap. Besides the link between cognitive flexibility and the level of autistic traits, there is evidence suggesting that performance in cognitive flexibility is a potential predictor of autism symptomatology in children (Kenworthy et al., 2014). Autism symptomatology scores could be related to cognitive flexibility scores, leading to a higher likelihood of decrease in autism symptomatology for individuals with greater cognitive flexibility. The current study aims to investigate the relationship between autism symptomatology and cognitive flexibility in both monolingual and bilingual autistic children.

### Bilingualism and Cognitive Flexibility in Autism

Research has shown that bilinguals outperform monolinguals on a range of EF tasks of a nonverbal nature (e.g., Bialystok et al., 2006; Paap et al., 2018). All speakers must resolve competition arising within the linguistic system. Such competition requires cognitive control processes to resolve conflict (Hofweber et al., 2021; Pivneva et al., 2012). Bilingual speakers face additional demands: They possess a language system optimized to handle two coding systems, and to use one language, they must control or inhibit the non-target one. The Adaptive Control Hypothesis (D. W. Green & Abutalebi, 2013) and the Control Process Model (D. W. Green & Li, 2014) have proposed that the use of the two languages within the same context (the dual-language context) provides strong advantages in EF for bilinguals. Though not consistently found across studies (see van den Noort et al., 2019 for a review), bilingualism effects have been mainly found in the cognitive subdomains of inhibitory control, decision-making, attention, and memory processing (Bialystok et al., 2004), as well as in set-shifting skills, with the latter being equated with cognitive flexibility (Bialystok & Viswanathan, 2009).

The dynamics of how bilingualism affects EF in autism are still poorly understood. Besides studies that have indirectly compared monolingual and bilingual autistic children on EF through parent report measures (Iarocci et al., 2017; Ratto et al., 2020), few studies provide direct evidence for the way bilingualism modulates autistic children’s EF performance (Gonzalez-Barrero & Nadig, 2019; Peristeri et al., 2020; Sharaan et al., 2020). More specifically, Iarocci et al. (2017) report on the results of an EF parental questionnaire that indexed school age and adolescent children’s ability to plan, anticipate, and maintain goal-directed behavior. Though the parents of the bilingual autistic children did not report any detrimental effect on their children’s cognitive skills, the bilingual autistic children experiencing EF deficits in the clinical range were significantly fewer relative to their peers having no second language exposure. Also, parents of bilingual autistic children in Ratto et al.’s (2020) study reported fewer cognitive flexibility and impulsivity deficits for their children as compared to the self-reports of parents of monolingual autistic children. In the same study, bilingual autistic childrens’ reduced cognitive flexibility problems were strongly associated with their scores on the restricted/repetitive patterns of behaviors and interests subscale measured through the Social Responsiveness Scale (Constantino & Gruber, 2012).

To the best of our knowledge, three studies provide direct evidence for the relation between bilingualism and cognitive flexibility in autistic children. Sharaan et al. (2020) did not find bilingualism effects in the performance of autistic children in nonverbal tasks tapping into flexible switching and interference control; yet, the bilingual autistic children outperformed their monolingual peers and even TD peers (both monolinguals and bilinguals) on a sustained attention task. Also, bilingual autistic children in Peristeri et al.’s (2020) study outperformed their monolingual peers (both TD and autistic) on a 2-back task with digits by making significantly fewer false hits. Crucially, Gonzalez-Barrero and Nadig (2019) were the first to show that bilingualism improves the set-shifting abilities of school-age autistic children relative to a group of age-matched monolingual autistic children.

One should mention, though, that the advantages reported for these studies refer to one outcome variable of the EF measure used. For instance, while Gonzalez-Barrero and Nadig (2019) found a positive effect of bilingualism on autistic children’s set-shifting skills, the opposite effect was found for working memory in daily life. Similarly, Sharaan et al. (2020) found that bilingualism positively affected autistic children’s sustained attention, but not their flexible switching and interference control skills. As such, it remains unclear whether a bilingual advantage may generalize to autistic populations and which factors modulate its strength. To the extent that the few studies that have investigated the effect of bilingualism in cognitive flexibility in autism show conflicting evidence (i.e., bilingualism either boosts cognitive flexibility or has no detrimental effects), further research is needed to make progress in understanding the interface between bilingualism and cognitive flexibility in autistic individuals. Another issue to consider is that previous studies have mainly employed nonverbal/visual tasks. One potential confound with such nonverbal measures is that they rely on visuospatial components of processing which are relatively intact in autism (Campbell et al., 2017; Kunda & Goel, 2011). Although there are good reasons to employ visuospatial set-shifting tasks as measures of cognitive flexibility in bilingual autistic children, it would be beneficial to explore the interplay between bilingualism and cognitive flexibility in language when separate tasks that require independent language processing are performed in a concurrent fashion.

### The Current Study

As previously discussed, research indicates cognitive flexibility difficulties for autistic individuals, while bilingualism may either carry advantages to this aspect of cognition (Gonzalez-Barrero & Nadig, 2019; Peristeri et al., 2020) or confer no benefits at all (Iarocci et al., 2017). These results need to be interpreted with caution given that bilingualism effects have been investigated indirectly through parent report measures (Iarocci et al., 2017), while the sample sizes were rather small (10 monolingual and 10 bilingual autistic children compared to 10 monolingual and 10 bilinguals TD peers in Gonzalez-Barrero & Nadig, 2019). Further research with larger sample sizes is necessary to verify whether bilingualism can mitigate flexibility difficulties in autistic children.

The current study aimed to investigate bilingualism effects in autistic children’s performance in three verbal dual-tasks that tapped into cognitive flexibility skills. In the first task, namely the listening span paradigm (Chrysochoou, 2006; Daneman & Carpenter, 1980), children listened to sentences, verified their semantic plausibility (truth/falsity), and then had to recall the individual words at the end of each sentence set. In the syntactic interference word recall task, children were asked to listen to sets of sentences of varying degrees of structural complexity (i.e., active transitives, passives, subject- and object-relatives), select the image that best matched the sentence out of multiple-choice visual arrays, and recall separate words that were presented after each sentence (Ivanova & Hallowell, 2014; Peristeri & Tsimpli, 2010). In the third task, namely the proactive interference task, children were presented with four sequential lists of words. Items in the first three lists were members of the same semantic category (fruit), while the words in the fourth list were members of a different semantic category (sports). After the presentation of each list and following a delay period during which the participant was instructed to count forward from a given digit for 10 s, the child was asked to recall the words of each list. As such, the first and the second dual-task tested the extent to which semantic and syntactic processing as secondary tasks, respectively, would interfere with word recall, which was the primary task of both dual paradigms; the third task, namely, the proactive interference task, tested the children’s susceptibility to interference of previously presented items belonging to the same semantic category, while counting aloud was manipulated in the task as an interfering foil.

The study’s research questions and hypotheses regarding autistic children and TD children are the following:

*Question 1*. What is the effect of bilingualism on autistic and TD children’s performance in the three dual-task paradigms? Are bilingual autistic and TD children more able to flexibly switch between the tasks in each dual paradigm as compared to their monolingual autistic and TD peers? Are bilingualism boosts in flexibility modulated by the different language demands across the three dual-tasks?
*Hypothesis 1*. Based on previous research showing that bilingualism improves EF in autism (Gonzalez-Barrero & Nadig, 2019; Iarocci et al., 2017; Peristeri et al., 2020; Ratto et al., 2020; Sharaan et al., 2020), we hypothesized that the bilingual autistic and TD children would perform better at the dual-tasks as compared to their monolingual peers. This effect was expected to manifest in autistic and TD bilingual children’s higher scores in word recall, which was the primary task of all three dual-task paradigms.

Regarding the extent to which the groups’ performances in the three dual-tasks would be modulated by the language demands in each task, we hypothesized that the bilingual children would be more flexible than their monolingual peers as a function of the language complexity in each dual-task. More specifically, the syntactic interference word recall task was expected to be more difficult than the listening span and the proactive interference task because of the syntactic processing of sentences with different levels of complexity that was expected to require a high working memory load. Syntactic impairments have often been reported for individuals on the autism spectrum (Kjelgaard & Tager-Flusberg, 2001; Tager-Flusberg, 2016) in contrast to semantic abilities, which have been claimed to be relatively intact (Eigsti et al., 2011; Tager-Flusberg et al., 1990). On the other hand, both the listening span and the proactive interference task include semantic processing, namely, sentence recall and recall of items belonging to the same semantic category, respectively. While semantic interference is involved in both tasks, the level of interference in the proactive interference task was expected to be higher than the listening span task, since the target responses (i.e., the words to-be-recalled) in the proactive interference task are the same with the units that need to be processed (i.e., the lists of words overlapping in terms of semantic categorical similarity). On the other hand, in the listening span task, it is sentences that need to be processed and judged, but the target is word recall.

*Question 2*. To what extent is the potential cognitive flexibility boost in bilingual autistic children modulated by individual differences?
*Hypothesis 2*. Based on previous findings that autistic traits, autism symptomatology, and stereotyped and rigid behaviors affect cognitive flexibility (Albein-Urios et al., 2018; Gökçen et al., 2014; Kenworthy et al., 2014; Ridley et al., 2011), we expected autistic children’s performance in the dual-tasks to be modulated by individual variation in age, SES, IQ, autism symptomatology (for the autistic children only), EF, language ability, and dual-language experience (for bilingual autistic and TD children only). We expected these features to affect children’s performance in the dual-tasks.

## MATERIALS AND METHODS

### Participants

The study included 200 children in total; 50 monolingual autistic children (ASDmono), 50 bilingual autistic children (ASDbi), 50 TD monolingual children (TDmono), and 50 TD bilingual children (TDbi), ranging in age from 7 to 12 years. The autistic children were recruited from schools and from public and private diagnostic centers in Greece. They had received a diagnosis of autism from a licensed child psychiatrist or a developmental pediatrician according to the standard diagnostic criteria (DSM-V; American Psychiatric Association, 2013). We confirmed the diagnosis of children with autism using the Autism Diagnostic Interview—Revised (ADI-R; Rutter et al., 2003). ADI-R total scores, as well as scores from the reciprocal social, verbal communication, and restricted and repetitive behaviors domains were used in the study as indicators of autism symptomatology (Coutanche et al., 2011; Hus & Lord, 2014; Meilleur & Fombonne, 2009; Uddin et al., 2011). At the group level, the autistic children had a mean full scale IQ score within the normal range (i.e., full IQ > 70; see, e.g., Bal et al., 2021; Botting & Conti-Ramsden, 2003; Koyama et al., 2007; Siegel et al., 1996) as measured through the Greek version of the Wechsler Intelligence Scale for Children (WISC-III; Wechsler, 1991; adapted to and standardized in Greek by Georgas et al., 2003); yet, 8 autistic children had an average verbal IQ (>70) and a below average performance IQ score (<70), while 4 autistic children exhibited the opposite pattern.

Typically developing children were recruited from public schools in Greece. The parents who provided written consent for the children’s participation in the study completed a questionnaire that included demographic information. The questionnaire also asked parents to report any family history of learning disabilities and their perception of any areas of difficulty for their child. Only children whose parents did not report any history of language difficulties and cognitive deficits participated in the study. This profile was also confirmed by the teachers’ written reports on the children’s academic achievements. Typically developing children were also administered the Raven’s Coloured Progressive Matrices (Raven et al., 1992). Both TDmono and TDbi children performed at a comparable level and within the normal range, as expected for their age.

**Figure 1.**
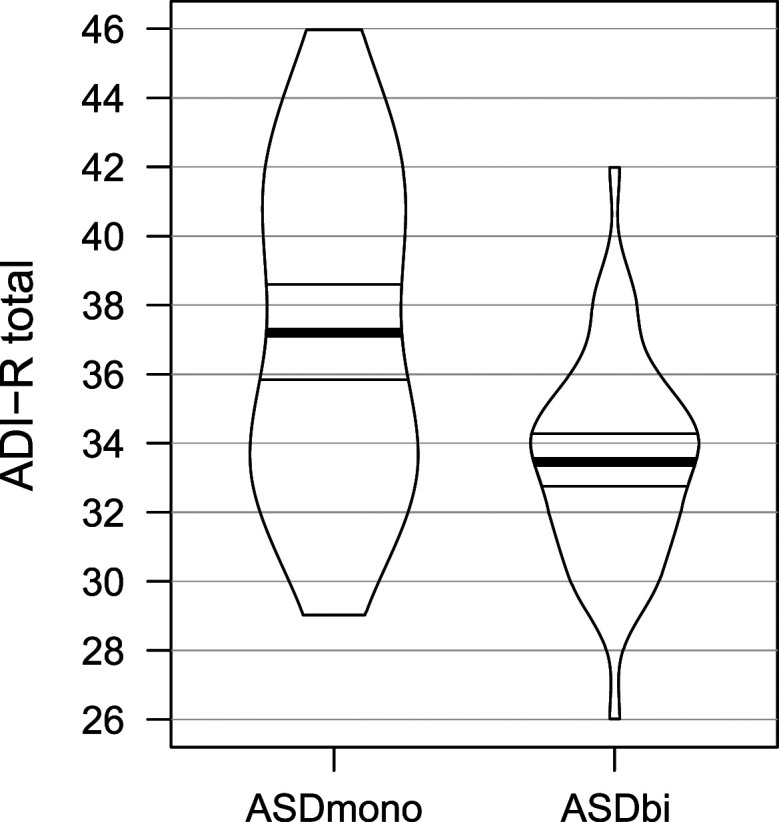
Distribution of the ADI-R total scores for the monolingual (ASDmono) and the bilingual (ASDbi) autistic groups.

Details of participant demographic characteristics are presented in Table 1. Descriptives were calculated using SummarySE (part of the Rmisc package; Hope, 2013) within the statistical analysis software R (version 3.6.2; R Core Team, 2019), and reported as means and standard deviations. *T* tests were performed to examine whether the groups differed in age, SES, autism symptomatology (only for the autistic groups), IQ, home language history (only for the bilingual groups), and current language use (only for the bilingual groups). In addition, *t* tests were used to compare the groups’ performance on two EF and two language ability measures. All *t* tests were performed as unpaired two sample Welch *t* tests and are reported with uncorrected *p* values, as their aim was to flag any potential between-group differences. Figure 1 illustrates the density of individual scores per group on the total autism symptomatology score of the ADI-R.

**Table 1.** Demographic information.

	TDmono	TDbi	ASDmono	ASDbi
*N*	50	50	50	50
Gender (M/F)	38/12	40/10	40/10	38/12
Age range	9.68 (0.94) 7.2–10.9	9.84 (1.11) 6.7–11.1	9.94 (1.46) 6.7–11.8	9.66 (1.61) 7.0–12.0
SES range	9.92 (2.53) 3–14	9.76 (2.29) 6–14	9.84 (2.19) 6–12	9.04 (3.10) 3–14
*IQ measures*				
VIQ (max. score: 160) range	–	–	89.30 (16.94) 50–142	90.32 (13.14) 70–109
PIQ (max. score: 160) range	–	–	88.58 (16.19) 66–133	90.96 (18.76) 49–127
IQ (max. score: 160) range	–	–	88.06 (16.16) 63–135	89.12 (14.14) 64–117
Raven (max. score: 36) range	26.2 (2.34) 20–30	26.6 (2.34) 16–31	–	–
*Executive function measures*				
loc-to-glo range	3.66 (12.11) −13–60	7.72 (6.99) −7–20	48.68 (25.87) 14–85	8.70 (12.94) −16–29
glo-to-loc range	24.70 (32.29) −4–94	19.19 (16.46) 3–114	11.24 (13.68) −8–57	26.12 (20.24) 0–63
*Language ability measures*				
Expressive vocabulary (max. score: 50) range	39.78 (6.33) 21–47	34.72 (5.34) 24–45	37.94 (6.28) 15–47	34.12 (6.74) 20–45
Sentence repetition (max. score: 96) range	65.92 (17.40) 32–92	63.61 (19.71)^a^ 26–90	63.36 (18.89) 29–95	59.40 (20.97) 10–89
*Bilingualism measures*				
Home language history (%) range		42.80 (21.22)^a^ 10–74		38.76 (11.86) 20–62
Current language use (%) range		45.39 (12.82)^a^ 29–67		49.04 (9.19) 30–67
*ASD symptomatology (ADI-R)*				
SOC (cutoff score: 10) range			21.02 (4.16) 14–29	17.54 (1.94) 14–23
VC (cutoff score: 8) range			12.10 (1.63) 9–15	12.16 (1.27) 9–14
RRB (cutoff score: 3) range			4.08 (0.97) 2–6	3.92 (9.66) 2–6
ADI-R total range			37.20 (4.76) 29–46	33.46 (2.92) 26–42

*Note*. ^a^*N* = 49. The demographic information is presented as Mean (*SD*), and includes: gender, age, socioeconomic status (SES), IQ measures (VIQ = verbal IQ; PIQ = performance IQ; IQ = general IQ for the autistic children; Raven scores for the TD children), executive function measures (glo-to-loc = costs of switching from a global to a local task; loc-to-glo = costs of switching from a local to a global task), language ability measures, bilingualism measures (i.e., home language history and current language use in L1 [Albanian/Russian/Bulgarian]), and autism symptomatology measures (results from the Autism Diagnostic Interview—Revised [ADI-R]; SOC = reciprocal social, VC = verbal communication, RRB = restricted and repetitive behaviors). TDmono = typically developing monolingual children; TDbi = typically developing bilingual children; ASDmono = monolingual autistic children; ASDbi = bilingual autistic children.

The TD versus autism and monolingual versus bilingual groups did not differ significantly in age and SES (which was measured through the mother’s education, in years). No IQ difference was found either between ASDmono and ASDbi or between TDmono and TDbi groups based on *t* tests. The two autistic groups did, however, differ in their ADI-R scores, specifically on their reciprocal social scores (with bilingual children scoring lower than monolingual children with autism; *t* = 5.36; *df* = 69; *p* < 0.001), and consequently on the total ADI-R score (*t* = 4.74; *df* = 81; *p* < 0.001). This difference can be seen in more detail in Figure 1. Note that for the other two ADI-R measures, namely, verbal communication and restricted and repetitive behaviors, there were no significant differences between the scores of the two autistic groups.

Monolingual children spoke Greek. Bilingual children, with and without autism, came from mixed marriages, and they were exposed to both languages from the time of birth. Thus, they were simultaneous bilinguals. The overwhelming majority of the children were Albanian-Greek speakers (11 bilingual children were Russian-Greek or Bulgarian-Greek speakers) and they were all dominant in Greek, which was the language used in school and often in the home context. The children’s bilingual experience was based on a comprehensive parental language history questionnaire, which asked about the home language history (i.e., the child’s exposure to each language from birth up to the age of four) and current language use (i.e., literacy and language preference in everyday life with family members or friends) of the child (Andreou, 2015). Following Andreou’s (2015) scoring of the questionnaire data, we awarded points for input in each language. Points were accumulated based on the number of people interacting with the child at different stages of development. For example, for home language, one or the other language was given 1 point, depending on whether a certain family member interacted with the child (father, mother, siblings, grandparents, etc.) in Greek or Albanian/Russian/Bulgarian, respectively. If a person interacted with the child in both languages, the point was divided between the two languages (0.5 points each). This score was normalized (in percentage) for the total number of people interacting with the child (in one language or another). Table 1 presents the home language history and current language use percentages for the Albanian/Russian/Bulgarian language. Implicitly, the remaining percentages correspond to the Greek language. The two bilingual groups did not differ significantly in home language history and current language use based on *t* tests.

The study was approved by the Research Ethics Committee of the Greek Ministry of Education, and parental consent was required for participation in the study.

### Procedure

Tests were presented in a fixed order, and scored according to standardized criteria. All the children completed two language ability tasks (expressive vocabulary, sentence repetition), an EF task (global-local cognitive flexibility), and three dual-task paradigms (listening span, syntactic interference word recall, proactive interference) during three sessions in a quiet room at the children’s home or school setting. All assessments were administered in Greek.

#### Expressive vocabulary task (Vogindroukas et al., 2009; adaptation from Renfrew, 1997)

The children’s expressive vocabulary in Greek was assessed through an expressive vocabulary test, which has been standardized for 3- to 10-year-old Greek-speaking monolingual children. It includes 50 black-and-white pictures of common objects that each child was asked to name individually. Each correct answer earns one point, with a maximum score of 50. The test was terminated when the participant failed to respond correctly to five consecutive trials.

#### Sentence repetition task

This task was developed within the Cost Action IS0804, *Language Impairment in a Multilingual Society; Linguistic Patterns and the Road to Assessment* (Chondrogianni et al., 2013).

##### Stimuli.

It includes 32 Greek sentences of varying degrees of syntactic complexity, such as coordination and subordination. Subordinate clauses include adverbial, complement, and relative clauses. Examples of a complement and a relative clause of the task are illustrated in (1) and (2) below.

##### Procedure.

Children sat in front of a computer screen and listened to the sentences via headphones. All sentences were recorded by a female native speaker of Greek and had normal intonation. An attempt to repeat a sentence was considered to be correct if it was a verbatim repetition of the target sentence. Children performed 5 practice trials and then the 32 experimental sentences.

##### Scoring.

Children’s performance on the sentence repetition task was scored for overall accuracy (Marinis & Armon-Lotem, 2015; Semel et al., 2003). More specifically, they were awarded a score of 3 if they made no errors while repeating the sentence, a score of 2 if they made one error, a score of 1 if they made two errors, and zero points if they made three or more errors.

(1)  i nosokomes ipan oti i ptisi tu jiatru ehi kaθisterisi.
    The nurses._NOM.FEM.PL_ said._PERF.INDIC.3P.PL_ that the flight._NOM.FEM.SG._ of the doctor._GEN.MASC.SG_ has._IMPERF.INDIC.3P.SG._ delay._ACC.FEM.SG_
    “The nurses said that the flight of the doctor is delayed.”
(2)  i kaθaristria klotsise tin nosokoma pu vjike apo to γrafio.
    The cleaning-lady._NOM.FEM.SG_ kicked._PERF.INDIC.3P.SG_ the nurse._ACC.FEM.SG._ that got._PERF.INDIC.3P.SG_ out of the office._ACC.NEUT.SG_
    “The cleaning-lady kicked the nurse that got out of the office.”

#### Global-local cognitive flexibility task

To assess the children’s ability to shift their attention to different levels of compound shape stimuli, we utilized a global-local cognitive flexibility task. The specific task was run on a computer with E-Prime (Schneider et al., 2002) and was based on a Navon-type letter task (Navon, 1977) that required participants to shift attention between global and local levels of a visual stimulus. The particular task was chosen among other EF paradigms, since it is a task in which participants are expected to show flexible behaviors (Ionescu, 2012). It can thus be treated as a nonverbal measure of cognitive flexibility.

##### Stimuli.

**Figure 2.**
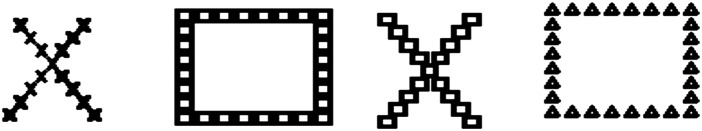
Example stimuli from the global-local cognitive flexibility task. The two shapes on the left are congruent trials, while the two shapes on the right are incongruent trials. The scale from local to global was 1:10. (Figure 2 is from Peristeri et al., 2021).

Four different shapes were used, namely, circles, Xs, triangles, and rectangles. Large shapes (global level) were composed of small shapes (local level). To present two conditions, large shapes were constructed from either identical small shapes (congruent condition; e.g., a big triangle made up of smaller triangles) or different shapes (incongruent condition; e.g., a big triangle made up of small Xs; see Figure 2).

##### Procedure.

The task of the children was to identify the number of lines required to form each shape (i.e., one line for circles, two for Xs, three for triangles, and four for rectangles) by pressing one of the four numbers (1, 2, 3, or 4) on the keyboard. The task comprised three building blocks, namely, the Global-No Shift, in which participants were asked to continuously respond to the global shape, the Local-No Shift, in which participants were asked to continuously respond to the local shape, and the Shift block, in which participants had to interchangeably respond to the global and local shape (global, local, global, local, etc.). Each target stimulus was presented on the screen until the participant responded. Children performed 8 practice trials and then 144 experimental trials. After completion of each block of 48 trials, each child was allowed a 5-minute break before completing the next block of the task.

##### Scoring.

For the purposes of the present study, we used data from both the Shift and the No Shift blocks to measure shifting costs. More specifically, we calculated local-to-global cognitive flexibility cost in the accuracy measure by subtracting accuracy scores in the local-to-global trials of the Shift block from accuracy in the global trials of the Global-No Shift block. Conversely, global-to-local cognitive flexibility cost in accuracy was calculated by subtracting accuracy scores in the global-to-local trials of the Shift block from accuracy in the local trials of the Local-No Shift block. The global-to-local and local-to-global shifting costs reflect global-to-local and local-to-global interference, respectively.

#### Dual-task paradigms: Listening span task

The listening span task was adapted from Chrysochoou (2006).

##### Stimuli.

The task included sentences arranged in separate blocks, beginning with single sentences and ending with six-sentence blocks in ascending order. Each subsequent block increased by one sentence. All of the sentences included four to six words. All of the sentence-final words were mono- or bisyllabic, high frequency, phonologically simple nouns and adjectives. Approximately equal numbers of nouns and adjectives appeared in sentence-final position across the sentences. Half of the sentences were semantically plausible (e.g., *The sky is pink*.), while the rest were nonsensical (e.g., *Houses can laugh*.). Table 2 presents the experimental items in the single- and the two-sentence blocks of the listening span task.

**Table 2.** Stimuli in the single- and the two-sentence blocks of the listening span task.

Level	Sentences	True/False	Words to-be-recalled
1	Scissors cut paper.	True	paper
	Goldfish have hair.	False	hair
	Lemons are sour.	True	sour
	Spiders make webs.	True	webs
	Clocks eat apples.	False	apples
	Giants are short.	False	short
2	Oranges live in the water.	False	water
	Roses smell sweet.	True	sweet
	Chairs give birth to eggs.	False	eggs
	Bananas have teeth.	False	teeth
	We wear shoes on our feet.	True	feet
	Apples grow on trees.	True	trees
	Humans have ears.	True	ears
	Oranges play the piano.	False	piano
	Fingers are on our hands.	True	hands
	Sheep have wings.	False	wings
	Cats work at schools.	False	schools
	Pigs have spun tails.	True	tails

##### Procedure.

Children listened to the prerecorded sentences, verified their semantic plausibility (truth/falsity), and were then asked to recall the individual words at the end of each sentence set. More specifically, the child was told that they will hear a female voice uttering sentences and then would need to do two things. First, respond “Yes” or “No” as to whether the event that the sentence describes could happen in real life and then recall as many of the sentence-final words at the end of each sentence block as they could remember. Serial order recall was not required. To pass on to the next sentence block, the child had to succeed in four-word recall trials. Responses were recorded by the examiner on an answer sheet.

##### Scoring.

According to the standard scoring procedure (Daneman & Carpenter, 1980), a score of 6 was awarded if the child made four correct successive final-word recall attempts, a score of 5 if one error is made, and a score of 4 if two errors are made independently of the child's accuracy performance in semantic plausibility judgment. If three errors were made, testing ceased. Nevertheless, to effectively capture interference from semantic plausibility judgment in word recall, we have only included in the word recall measure the items of the trials in which the child’s semantic plausibility judgment was correct. The outcome measures were: the final-word recall score, the number of the sentence-blocks that the child succeeded in passing on to, and the semantic judgment accuracy score. The maximum final-word recall score was 36, while the maximum score for the sentence-blocks was 6 points. Internal consistency reliability coefficients for the composite score ranged from 0.89 to 0.94 (Median  =  0.93).

#### Dual-task paradigms: Syntactic interference word recall task

##### Stimuli.

**Figure 3.**
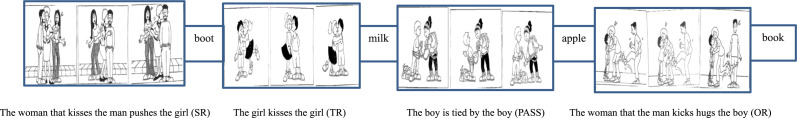
Example stimuli of a sentence-picture matching and a word recall block of the syntactic interference word recall task. SR = subject-relative; TR = active transitive; PASS = passive; OR = object-relative.

The task included four blocks of sentences, each including one active transitive, one passive, one subject-relative, and one object-relative sentence. There were 16 sentences in total. All the sentences included animate agents and patients and as such, they were semantically reversible. Two- and three-syllable, high frequency nouns and verbs were used in the sentences. Each sentence was accompanied by a multiple-choice image array. Each array consisted of three pictures: one target and two foils. Each sentence block included four to-be-remembered words, which were two- or three-syllable nouns from different semantic categories; they did not rhyme with each other and were unrelated to the sentences. As such, each experimental block included four picture-matching trials and four words to-be-recalled. The position of the sentences in each block and the location of the target in each image array were counterbalanced across the four blocks. An example of a block of the syntactic interference word recall task is presented in Figure 3.

##### Procedure.

The task was run on PowerPoint (Microsoft Corporation). Children were asked to listen to the blocks of sentences, select the picture that best matched the meaning of each sentence, and recall the words that popped up between the sentence-picture matching trials. More specifically, along with the auditory presentation of each sentence, the multiple-choice image array was presented. The child was asked to point to the picture that best matched the meaning of the sentence. After each sentence-picture matching trial, a word was auditorily presented and displayed on the center of the screen. The child was asked to remember the word so that they could recall it later on. After the fourth word was auditorily presented and displayed on the computer screen, a blank screen appeared and the child was asked to recall aloud as many of the words as they could remember. Responses were recorded by the examiner on an answer sheet.

##### Scoring.

The dependent variables were: the word recall score and the sentence-picture matching accuracy score (Peristeri & Tsimpli, 2010). The child was awarded 1 point for each correct sentence-picture matching trial and 1 point for each successful word recall trial. The maximum score for both sentence comprehension and word recall was 16 points each.

#### Dual-task paradigms: Proactive interference task

The proactive interference task of the present study was modelled after Bialystok and Feng’s (2009) study.

##### Stimuli.

There were four lists of words containing seven words each. The words in the first three lists belonged to the same semantic category (i.e., fruit), while the words in the final/fourth list belonged to sports.

##### Procedure.

Experimental stimuli were displayed on a monitor controlled by E-prime software (Schneider et al., 2002). Children first viewed a red light warning signal (“Get ready for the first List”) against a white background for 2 s, after which the words from the first list appeared one at a time. The words across the four lists appeared in black small letters against a white background. Following Hamilton and Martin’s (2007) paradigm, each of the seven words was presented for 1,000 ms, separated by a 100 ms inter-stimulus interval. Auditory files of the word stimuli were recorded as WAV files and each word was auditorily presented through computer speakers as soon as it was displayed on the screen. Immediately after the final word in each list disappeared, a random number between 19 and 22 appeared on the screen and the participant was orally instructed to count forward from the specific number for 10 s. After the child counted for 10 s, a blank screen appeared and an oral instruction cued them to orally recall the words from the list. Children had 20 s to recall the words in any order and were encouraged to keep working to recall throughout the entire period. Responses were recorded by the examiner on an answer sheet. At the end of the recall period, a screen appeared for 2 s, which instructed the children to stop recalling. The same sequence, beginning with the red warning signal, was repeated for lists 2, 3, and 4.

##### Scoring.

The mean number of words that had been accurately recalled per list was recorded on an answer sheet and counted for each child. Proactive interference effects were subsequently calculated for lists 2 and 3 by subtracting the number of words recalled on list 1 from the number of words recalled on lists 2 and 3.

### Analyses

The experimental tasks were coded for analysis. All analyses were performed within the statistical analysis software R (version 3.6.2; R Core Team, 2019). First, we present the results of the Welch *t* tests, which were used to compare the groups’ performance on the two language ability measures (i.e., expressive vocabulary and sentence repetition), and the global-local cognitive flexibility task. Linear regression analyses were then carried out to examine the influence of bilingualism, autism, age, SES, autism symptomatology/ADI-R totals (only for the autistic groups), IQ, home language history (only for the bilingual groups), current language use (only for the bilingual groups), EF (global-local cognitive flexibility and local-global cognitive flexibility), and language ability (expressive vocabulary and sentence repetition) as independent variables on word recall as the dependent variable in each task. Independent variables that did not reach significance were retained in the models so that the output of the different models is maximally comparable. Uncorrected *p* values are reported throughout the article and in the Supporting Information (located at https://doi.org/10.1162/nol_a_00055).

In addition, only for the syntactic interference task, overall sentence comprehension was examined as a dependent variable to determine the groups’ syntactic abilities. Separate models were developed for each of the dependent variables. Treatment coding was used for the groups (bilingualism and autism); all other dependent and independent variables were numeric. Separate models were performed for the TD and the autistic groups in order to get a full overview of the factors influencing the performance of the different groups. This could be seen as an alternative to testing for interactions, which, with so many factors, would quickly become difficult to interpret. In addition, testing the groups in pairs eliminates the need for the post hoc testing of any main effects of group (e.g., through follow-up pairwise *t* tests). Thus, the choice of running models separately for the TD and the autistic children was made to increase the interpretability of the analyses as well as to remove the need for follow-up, post hoc comparisons. The full model output can be found in the Supporting Information.

To correct for multiple comparisons, the false discovery rate controlling procedure (Benjamini & Hochberg, 1995) was used. More specifically, we corrected for the fact that multiple tests were used to investigate the two main variables of interest, namely bilingualism and autism. In total, 10 different tests examined the effects of bilingualism and 10 different tests examined the effects of autism. The false discovery rate controlling procedure corrects the significance thresholds of those tests from the typical α = 0.05 threshold to a lower threshold of α = 0.05*i*/*n*, with *i* being the comparison number based on the ordered *p* values and *n* being the total number of comparisons for a variable of interest (10 in this case; for more details, see Benjamini & Hochberg, 1995). Cases in which the significance after corrections for multiple comparisons differs from the significance before corrections will be mentioned explicitly in the text.

Finally, the standardized effect sizes of the significant effects of bilingualism in autism for each of the dual-task paradigms were calculated using Cohen’s *d* (with effect sizes of *d* < 0.5 being small; *d* < 0.8 being medium; otherwise being large; Cohen, 1992).

## RESULTS

### Expressive Vocabulary and Sentence Repetition Tasks

Both groups of bilingual children showed lower expressive vocabulary scores than their monolingual peers (TD groups: *t* = 4.32; *df* = 95; *p* < 0.001, autistic groups: *t* = 2.93; *df* = 98; *p* < 0.01). No significant differences in the sentence repetition scores were found between the groups.

### Global-Local Cognitive Flexibility Task

On the EF measure, local-to-global cognitive flexibility costs did not differ significantly between the two TD groups, nor between the bilingual TD group and the bilingual autistic group. However, significant differences were found between the two autistic groups, with monolingual children with autism showing increased cognitive flexibility costs (*t* = 4.30; *df* = 86; *p* < 0.001), and between the two monolingual groups, again with monolingual autistic children showing increased cognitive flexibility costs (*t* = 2.71; *df* = 66; *p* < 0.01). Thus, monolingual autistic children seemed to show increased costs when switching from the local to the global level, whereas bilingual autistic children performed similarly to their TD peers. Interestingly, cognitive flexibility costs for switching in the other direction, namely from the global to the local level, showed the opposite effects: monolingual autistic children showed decreased costs compared to bilingual autistic children (*t* = 9.77; *df* = 72; *p* < 0.001) and monolingual TD children (*t* = 11.14; *df* = 70; *p* < 0.001).

### Dual-Task Paradigms

The results for the different dual-task paradigms are listed in Table 3. For each task, all measures are reported for completeness of information. However, only the word recall performance will be analyzed for all dual-task paradigms, as this is the primary task where interference from the secondary tasks is likely to occur. After discussing the general patterns in the results, namely, the effects of bilingualism and autism, we will additionally examine the groups’ syntactic abilities in the syntactic interference task more closely.

**Table 3.** Results for the three different dual-task paradigms.

	TDmono	TDbi	ASDmono	ASDbi
*Listening span task*				
Word recall	6.28 (2.78)	7.64 (3.27)	5.26 (2.26)	8.36 (3.78)
Block level	1.22 (0.76)	1.66 (0.80)	1.34 (1.14)	1.86 (0.97)
Semantic judgment	9.86 (3.45)	11.00 (3.94)	8.48 (4.73)	11.58 (4.73)
*Syntactic interference word recall task*				
Word recall	12.18 (2.00)	12.48 (1.61)	8.60 (2.78)	10.60 (3.52)
Recall after transitives	3.60 (0.67)	3.74 (0.49)	2.28 (1.07)	2.96 (1.05)
Recall after passives	2.66 (0.98)	2.70 (1.09)	2.22 (0.97)	2.24 (1.22)
Recall after subject relatives	3.00 (0.90)	2.98 (0.87)	2.08 (0.97)	2.76 (1.35)
Recall after object relatives	2.92 (0.88)	3.06 (0.87)	2.02 (1.04)	2.64 (0.85)
Sentence comprehension	12.12 (2.29)	10.20 (2.36)	9.28 (2.75)	9.52 (2.71)
*Proactive Interference task*				
Score list 1	5.18 (0.66)	5.48 (0.79)	4.58 (1.11)	4.78 (1.18)
Score list 2	4.06 (1.22)	4.26 (1.37)	2.26 (1.10)	4.08 (1.37)
Score list 3	3.80 (1.21)	4.16 (1.33)	1.72 (0.73)	3.74 (1.69)
Score list 4	5.86 (0.61)	6.12 (0.80)	5.10 (1.05)	5.74 (1.01)
Proactive interference list 2	−1.12 (1.08)	−1.22 (1.27)	−2.32 (1.28)	−0.70 (1.28)
Proactive interference list 3	−1.38 (1.12)	−1.32 (1.24)	−2.86 (1.29)	−1.04 (1.71)

*Note*. The results are presented as Mean (*SD*). TDmono = typically developing monolingual children; TDbi = typically developing bilingual children; ASDmono = monolingual autistic children; ASDbi = bilingual autistic children.

### Effects of Bilingualism

The first set of regression analyses examined whether the interference effect in the dual-task paradigms is attenuated by bilingualism in autistic children and TD children. To this end, the two TD groups (monolinguals and bilinguals) were analyzed separately from the two autistic groups (monolinguals and bilinguals). The full model results can be found in the Supporting Information.

On word recall in the listening span task, the results from the TD groups show no significant difference between the performance of TD bilingual children and TD monolingual children, although a trend of TD bilingual children performing better than TD monolingual children was observed (*p* = 0.06). Only sentence repetition significantly correlated with performance on word recall for the TD children (*p* < 0.01). On the same task, the results from the autistic groups show that bilingual autistic children outperformed monolingual autistic children (*p* < 0.001; effect size *d* = 1.03 (large), CI [0.61, 1.45]) indicating that bilingualism can attenuate language interference effects in a word meaning-based task in autistic children. The results from the autistic groups further show positive effects of EF (both global-local and local-global switching costs; *p* < 0.01).

On word recall in the syntactic interference word recall task, no significant effect of bilingualism was found for the TD children, although a trend was observed (*p* = 0.07). In contrast, significant effects of age (*p* < 0.05) and sentence repetition were found (*p* < 0.05). For the autistic children, the effect of bilingualism did reach significance, with bilingual autistic children outperforming their monolingual peers (*p* < 0.05; effect size *d* = 0.63 (medium), CI [0.22, 1.04]). In addition, an effect of expressive vocabulary score was found, indicating that children who performed better on this measure of general language ability scored better on word recall in the syntactic interference word recall task (*p* < 0.01).

Finally, on the proactive interference task, we analyzed the proactive interference on lists 2 and 3 compared to list 1. For the TD children, no significant effects of bilingualism were found on list 2 or list 3. On the proactive interference on list 2, only a positive effect of expressive vocabulary score was found (*p* < 0.05). On the proactive interference on list 3, no significant effects of any of the variables of interest were found. For the autistic children, a positive effect of bilingualism was found for list 2 (*p* < 0.05; effect size *d* = 1.26 (large), CI [0.83, 1.70]), indicating that bilingual autistic children performed better than their monolingual peers. Notably, the monolingual autistic children scored much lower on list 2 than the other three groups. On list 3, the monolingual children still scored lower, but the influence of bilingualism on proactive interference on list 3 did not reach significance in the regression model. Instead, an effect of EF (global-to-local switching costs) was found (*p* < 0.05).

Overall, effects of bilingualism were found for the autistic children on all three different dual-task paradigms. Note that the standardized effect sizes differed between the three different tasks: The sizes of the effects of bilingualism were large in the listening span and in the proactive interference task, whereas the effect size was medium in the syntactic interference word recall task. If we assume that the study has a sufficient sample size and sufficient power to draw conclusions from the observed effect sizes, we can conclude that the effect of bilingualism in autism in the syntactic interference task is smaller relative to the other two dual-task paradigms. Reasons for why this could be the case are discussed in the Discussion section. For the TD children, there were marginal effects of bilingualism on the listening span task and the syntactic interference task, but not on the proactive interference task.

### Effects of Autism

The second set of regression analyses examined the extent to which the performance of the TD bilingual children and bilingual autistic children in the dual-tasks is affected by home language history and current language use metrics. In addition, we compared dual-task performance effects in monolingual children with and without autism. To this end, the two monolingual groups (TD and autism) were analyzed separately from the two bilingual groups (TD and autism). The full model results can be found in the Supporting Information.

On word recall in the listening span task, the results from the monolingual groups show that TD children significantly outperformed autistic children (*p* < 0.01). In addition, EF skills significantly correlated with performance on word recall (*p* < 0.05); more specifically, local-global switching costs were found to be positively correlated (*p* < 0.05) with word recall. The results from the bilingual groups show, in contrast, no effect of autism, indicating that autistic children performed similarly to TD children. Effects of language ability (sentence repetition; *p* < 0.01) were found, with higher scores on sentence repetition correlating with better scores on word recall. Notably, no significant effects of home language history and current language use were found.

On word recall in the syntactic interference task, a significant difference was found between TD monolingual children and monolingual autistic children (*p* < 0.05), with the latter group underperforming. In addition, the analyses of the monolingual groups showed a negative effect of SES (*p* < 0.01) and a positive effect of sentence repetition score (*p* < 0.01). The results from the bilingual groups also showed a significant negative effect of autism (i.e., TD bilinguals outperformed those with autism; *p* < 0.01). In addition, a positive relation between expressive vocabulary and word recall in the syntactic interference task was found (*p* < 0.05). No other variables had a significant effect on bilingual children’s performance.

For the proactive interference task, we again analyzed the proactive interference on lists 2 and 3 compared to list 1. For the monolingual children, no significant effects of autism were found on list 2 or list 3 (although a trend was found for list 2, *p* = 0.06; for list 3, *p* = 0.10). On the proactive interference on list 2, no significant effects of any of the variables of interest were found. On the proactive interference on list 3, only a negative effect of local-to-global switching costs was found (*p* < 0.01). For the bilingual groups, the effect of autism on proactive interference on list 2 just reached significance (*p* < 0.05), implying that interference may be lower for bilingual autistic children compared to TD bilingual children. However, this effect loses significance when correcting for multiple comparisons. In addition, it should be noted that the bilingual autistic children overall scored lower on the recall task than TD bilingual children, but their performance decreased less when interference occurred. Other effects found for proactive interference on list 2 were local-to-global switching costs (*p* < 0.05) and expressive vocabulary (*p* < 0.05). For proactive interference on list 3, no significant effects of autism were found; only an effect of local-to-global switching costs (*p* < 0.05) was found.

### Syntactic Abilities in Monolingual and Bilingual Children With and Without Autism

A final set of regression analyses examined the groups’ syntactic abilities as displayed in the syntactic interference task more closely. The full model results can be found in the Supporting Information. On overall sentence comprehension scores, TD monolinguals outperformed TD bilinguals (*p* < 0.05), but autistic bilinguals outperformed autistic monolinguals (*p* < 0.05). In addition, in the autistic groups there were effects of age (negative, *p* < 0.05), EF (global-to-local switching costs, negative, *p* < 0.001), and language abilities (expressive vocabulary, positive, *p* < 0.001; sentence repetition, positive, *p* < 0.001). The TD groups did not show effects of EF or language abilities.

When comparing the two monolingual groups, TD monolingual children outperformed monolingual autistic children on sentence comprehension (*p* < 0.001). Again, a negative correlation with age was found (*p* < 0.05), as well as a positive correlation with sentence repetition (*p* < 0.01). Finally, in the bilingual groups no effect of autism was found. The bilingual groups only showed a significant correlation between expressive vocabulary (*p* < 0.01) and sentence comprehension in the syntactic interference task.

## DISCUSSION

The current study investigated bilingualism effects in the dual-task performance of autistic children and age- and SES-matched TD children. Our design hinges on a key feature of the cognitive profile in autism known as rigidity, which prevents individuals on the autism spectrum from efficiently regulating their behavior and allocating attention to more than one input stream in dual-task conditions (Geurts et al., 2009; Poljac & Bekkering, 2012; Schmitz et al., 2006; Yerys et al., 2015). Reduced cognitive flexibility often makes autistic individuals persevere on discrete and narrow information, struggling with transitions or not seeing novel relationships in multitasking situations (Bos et al., 2019; D’Cruz et al., 2013). Conversely, dual language exposure has been linked to advantages in nonverbal EF, especially in sustained attention (Sharaan et al., 2020), set shifting (Gonzalez-Barrero & Nadig, 2019), and inhibition (Peristeri et al., 2020). Experimental evidence of the effects of bilingualism on the cognitive flexibility of autistic children remains limited. This study aimed to explore whether bilingualism would compensate for reduced cognitive flexibility in autism with a focus on language, and more specifically, on dual-task conditions in which language processing in the form of semantic judgment, sentence parsing, and digit counting (as a secondary task) interfered with word recall (as the primary task). As such, the degree of interference across the dual-task paradigms varied based on the type of the language processing that needed to be performed during the secondary, interfering task. The results revealed that bilingual autistic children significantly outperformed their monolingual peers in the primary task (i.e., word recall) across all three dual-tasks, which suggests that bilingualism can mitigate cognitive flexibility deficits in autism. Crucially, autistic children’s performance in the dual-tasks was found to be modulated mainly by EF skills, which implies that autistic children relied more on EF rather than their language skills to cope with the dual-task demands.

Our first research question was whether bilingualism would affect TD and autistic children’s word recall performance in the dual-task paradigms. Bilingual autistic children scored higher than their monolingual peers in all three dual-task paradigms (i.e., the listening span, the syntactic interference word recall, and the proactive interference task), while their TD peers showed no significant effects of bilingualism in any of the three dual-task paradigms. These findings suggest that bilingualism enabled autistic children to cope better than their monolingual peers with multitasking and the demands on distinct control processes that needed to be implemented, such as adaptive task control, interference detection and inhibition, and attention.

More specifically, in the listening span task, the bilingual autistic group outperformed the monolingual autistic group, while better word recall scores were found in the children experiencing higher costs of switching from global-to-local and local-to-global trials. Interestingly, monolingual autistic children exhibited a more local-level processing style in the global-local cognitive flexibility task in comparison to their bilingual peers. This effect was not due to outliers; in fact, there were multiple monolingual autistic children that showed very high local-to-global switching costs, and the highest costs (as well as the lowest ones) were within 2 standard deviations of the group’s mean. It is noteworthy that the same monolingual autistic children, who showed high local-to-global switching costs, exhibited lower costs when shifting from the global to the local level compared to their bilingual peers (see *t* test comparisons of the children’s scores on the global-local cognitive flexibility task in the Results section). This finding is in line with previous research (e.g., Happé & Frith, 2006; Muth et al., 2014) showing that autistic individuals demonstrate a unique detail-focused processing style at the expense of the larger picture. This discrepancy between the autistic groups’ performance in the global-local cognitive flexibility task is not necessarily an issue from a statistical point of view; in fact, to control for this difference, we have included the global-to-local and local-to-global measures in all the statistical models of the study. Furthermore, in the listening span task, there were no significant word recall differences between bilingual children with and without autism. In the syntactic interference word recall task, monolingual autistic children recalled significantly fewer words than both TD monolingual and autistic bilingual children.

In the proactive interference task, the expected decline in word recall between lists 1 and 3 was found across all the experimental groups. Children showed an increase in competition between the items to-be-recalled and the reactivated non-target items as the experiment progressed, with the decrease in word recall performance being the highest on list 3. Differences in the proactive interference build-up effect were most explicitly shown on list 2 of the task, in which monolingual autistic children scored lower, and thus, showed a sharper memory decline than bilingual autistic children. Whether the interference effect exhibited by the monolingual autistic group in the proactive interference task reflects the children’s inability to efficiently inhibit memory traces of previously presented items that persisted in memory or to a rapid decay of lexical representations cannot be conclusively determined. Christ et al. (2011) have also examined proactive interference effects in a group of autistic individuals ranging in age from 8 to 18 years; though the autistic participants scored lower than their TD peers on list 2 (compared to list 1) of the task, the difference was not statistically significant. However, the proactive interference task in Christ et al.’s (2011) study included 4-item lists (vs. our paradigm that included 7-item lists), while the lexical items were presented in written form along with their corresponding images, which may have boosted autistic children’s memory retrieval capacity (Mostert-Kerckhoffs et al., 2015). The overall evidence from the dual-task paradigms of the present study demonstrates that bilingualism compensated for reduced cognitive flexibility in autism. The effect of bilingualism on autistic children’s cognitive flexibility profile is consistent with previous studies that have also found that bilingualism attenuates autism-related deficits in core cognitive areas, including theory of mind (Andreou et al., 2020), language adaptation to specific contexts (Yu, 2016), and narrative ability (Peristeri et al., 2020).

Our prediction about the way performance in the dual-task paradigms would be modulated by the language demands in each task was verified. Bilingualism effects for the autistic children were stronger in the listening span task as compared to the syntactic interference word recall paradigm. According to our initial prediction, semantic judgment in the listening span task was hypothesized to be a cognitively non-taxing secondary task which allowed bilingual autistic children to be more tuned into the primary task and thus recall more words than the monolingual autistic group, which scored significantly lower than TD controls. Monolingual autistic children’s poor word recall capacity in the listening span task seems to be associated with difficulties in resolving interference from the secondary, semantic plausibility judgment task. In this measure, accuracy scores of the monolingual autistic group were the lowest of all groups. It is therefore likely that semantic plausibility judgment had a greater interfering effect relative to the rest of the dual-task paradigms, which made it harder for the monolingual autistic children to retrieve the target final words of the sentences. Their low performance may be associated with the fact that sentences in the semantic judgment task required lexical semantics and, most importantly, world knowledge reasoning. More specifically, judging the plausibility of the sentences tapped into either broad world knowledge information about habitual events (e.g., *Spiders make webs*) or the borderline meaning of vague predicates such as relative adjectives (e.g., *Giants are short; Roses smell sweet*) that require a mental context for their interpretation. According to recent findings (Farmer et al., 2017; Howard et al., 2017; Saldaña & Frith, 2007), the indeterminacy of vague linguistic expressions whose meaning draws on context, and the integration of world knowledge in language comprehension can be problematic for autistic individuals, and this may explain the low performance of monolingual autistic children in semantic judgment.

Similarly, the bilingualism effect in autism was stronger in the proactive interference task relative to the syntactic interference word recall task. Specifically, in the proactive interference task, interference from non-target words sharing semantic categorical similarity may have penalized monolingual autistic children’s word retrieval capacities. Similarity-based interference has been widely acknowledged as a key factor that contributes to processing difficulty in language production and comprehension (Fukumura et al., 2011; Lewis & Vasishth, 2005; McElree, 2000), and may have also modulated the strength of the bilingualism effect on autistic children’s word recall performance in the current study. On the other hand, in the syntactic interference task, bilingual autistic children scored higher in word recall than their monolingual peers, with better word recall scores being reported for younger children experiencing less global-to-local interference, as well as children with better sentence repetition and expressive vocabulary skills. However, the effect size of the bilingualism effect in autism in the syntactic interference task was smaller relative to the other two dual-task paradigms. The regression analyses of the syntactic interference data show that bilingual autistic children’s lower scores in sentence repetition and expressive vocabulary as compared to the monolingual autistic group may have contributed to processing difficulty with sentence parsing, subsequently freeing up fewer cognitive resources for the retention in memory and the retrieval of the lexical items in the word recall component of the dual-task.

The study’s second research question focused on the role of individual differences on monolingual and bilingual autistic children’s performance in the dual-task paradigms with a particular emphasis on EF, age, SES, language ability, IQ, and autism symptomatology, as well as home language history and current language use for the bilingual children only. In the listening span task, EF effects were found to be mostly relevant to the autistic rather than TD children. More specifically, for the combined autistic groups, global-to-local and local-to-global interference affected their performance in the word recall component of the task, while TD children (monolinguals and bilinguals combined) seemed to rely more on their sentence repetition skills. As already mentioned, the listening span test found no significant behavioral differences between bilingual children with and without autism, whose performance was correlated with sentence repetition. EF effects seemed to be least influential in the syntactic interference task. Specifically, bilingual autistic children’s higher word recall performance than monolingual autistic children’s was associated with expressive vocabulary, while autistic children’s lower word recall performance than TD children’s was found to be associated with autistic children’s lower performance in sentence repetition and expressive vocabulary skills. Furthermore, in the proactive interference task, word recall on list 2 for the bilingual autistic group was higher for the children experiencing stronger local-to-global interference.

None of the regression analyses showed significant effects of autism symptomatology or IQ score for the autistic children. Therefore, it is unlikely that the differences between monolingual and bilingual autistic children in the dual-task paradigms are due to a difference in autism symptomatology. Though bilingual autistic children scored lower than their monolingual peers on autism symptomatology, and more specifically in reciprocal social skills, this difference was not reflected in the groups’ performance in the dual-task paradigms. Of course, this may be due to difficulty in finely quantifying autism symptomatology with the ADI-R tool; symptoms of autistic individuals are measured on a relatively coarse scale, which raises the possibility that this scale could not fully capture nuanced individual differences in autism-related symptomatology. Furthermore, there were no effects of age and SES on autistic children’s performance, and no separate influence of home language history or current language use for the bilingual children. However, it could be the case that these demographic measures were captured by other variables included in the models, such as EF and language abilities, and therefore failed to reach significance as a separate factor.

The extent to which bilingual autistic children’s cognitive flexibility skills map on brain functions remains unknown, as the underlying spatial and temporal dynamics of cognitive flexibility have not been studied yet in bilingual autistic populations. Studies that have explored the neural correlates of set-shifting performance in TD bilingual children converge to show that bilinguals outperform monolinguals, and that this flexibility advantage likely reflects enhanced left prefrontal and inferior frontal functions (Garbin et al., 2010; Moriguchi & Lertladaluck, 2020; Stocco et al., 2014), which have been reported to be recruited for language control in bilinguals (Calabria et al., 2012; Jasinska & Petitto, 2013). The specific findings imply that enhanced cognitive flexibility in TD bilingual children is supported by language-specific mechanisms, though there are studies that support the idea that bilingual language control is a subdomain of general executive control (Abutalebi et al., 2008, 2011; Craik & Bialystok, 2006; Garbin et al., 2010). The dissociation of the brain areas involved in set shifting across TD monolinguals and bilinguals, coupled with the atypical brain activation patterns in monolingual autistic children (Taylor et al., 2012; Yerys et al., 2015), makes accurate characterization of the brain network underlying cognitive flexibility in bilingual autistic children all the more challenging. Activation of left prefrontal and inferior frontal brain regions, which have been shown to be correlated with language control in bilinguals, may provide a mechanism for improved cognitive flexibility performance in bilingual autistic children as compared to monolingual autistic children. Neuroimaging can provide a means for understanding neurobiological differences between monolingual and bilingual autistic populations. Future directions of our research include attempts at investigating cortical brain volumes in monolingual and bilingual autistic children, and correlating these measures with behavioral outcomes in nonverbal and verbal cognitive flexibility tasks.

In the current study, we have compared monolingual and bilingual children with and without autism in terms of their performance in verbal dual-task paradigms, while also controlling for the children’s EF, autism symptomatology, IQ, SES, and dual-language experience. Bilingual autistic children outperformed their monolingual peers in all dual-task paradigms; yet, the bilingualism effect was stronger in the paradigms in which the secondary, interfering task drew on lexical semantics rather than syntactic information. Bilingual autistic children’s performance in the dual-task paradigms was mostly driven by their EF rather than their language ability. The overall evidence shows that bilingualism, in the form of enhanced language experience, compensates for reduced cognitive flexibility in language. This particular finding is of clinical importance, since cognitive flexibility permeates nearly every aspect of our lives and its disruption in autism results in profound life-altering deficits. An alternative explanation might be one of cognitive preservation, whereby bilingual autistic children may possess greater cognitive capacities in the face of increased demands, which could have helped them preserve both tasks in memory, allowing for word retention to occur for more time than it otherwise would be able to do (see Bak et al., 2014). However, studies of aging in autism as well as longitudinal studies are needed to further explore this possibility.

Although the results of the current study provide evidence for a positive effect of bilingualism on autistic children’s cognitive flexibility skills, our findings should be replicated by follow-up studies, given the following limitations. First, despite IQ scores not affecting autistic children’s performance in the dual-task paradigms, no direct comparisons were run between TD and autistic groups on IQ, which raises the possibility that the dual-task performance differences between groups might have been affected by IQ differences. Furthermore, the ADI-R tool may have lacked the nuance of specific inflexible behaviors, including rigidity, perseverative thought, and insistence on sameness found in autistic individuals, thus, failing to correlate with the dual-task outcomes of the current study. Other ratings of rigidity in autistic individuals’ daily life may likely be better predictors of the nuances involved in cognitive flexibility measures. The way that bilingualism effects on cognitive flexibility skills may transfer or generalize to the social competence of autistic individuals is an important question that needs to be explored in future research.

## ACKNOWLEDGMENTS

We are grateful to the children and the children’s parents for their unfailing commitment and interest in our study.

## AUTHOR CONTRIBUTIONS

**Eleni Peristeri**: Conceptualization: Supporting; Data curation: Lead; Funding acquisition: Lead; Investigation: Lead; Methodology: Lead; Project administration: Equal; Resources: Equal; Writing – original draft: Lead; Writing – review & editing: Equal. **Margreet Vogelzang**: Formal analysis: Lead; Software: Supporting; Visualization: Lead; Writing – original draft: Equal; Writing – review & editing: Equal. **Ianthi Maria Tsimpli**: Conceptualization: Lead; Methodology: Equal; Supervision: Lead; Writing – review & editing: Lead.

## TECHNICAL TERMS

Cognitive flexibility: Refers to the ability to adapt cognitive behavior to changing contexts.
Autism symptomatology: Refers to the severity of the symptoms in social and verbal communication, and restricted repetitive behavior domains.
