# Supplementary material for: Bilingualism Effects on the Cognitive Flexibility of Autistic Children: Evidence From Verbal Dual-Task Paradigms
Source: Neurobiol Lang (Camb). 2021 Dec 23;2(4):558–85. doi: 10.1162/nol_a_00055 (PMC10198706; doi:10.1162/nol_a_00055)
Supplement: Supplementary file 1 [file nol-2-4-558-s001.pdf]

Supplementary Materials

Bilingualism effects

Linear model results for listening span scores

TD groups:

	Estimate	Std. Error	t value	Pr(>|t|)
(Intercept)	0.49407	2.94548	0.168	0.86716
bilinguals	1.40751	0.72490	1.942	0.05527 .
age	0.13663	0.42246	0.323	0.74712
SES	0.05441	0.12509	0.435	0.66462
loc-to-glo	0.01241	0.03055	0.406	0.68550
glo-to-loc	-0.01728	0.01210	-1.429	0.15648
expressive vocabulary	0.01537	0.06864	0.224	0.82334
sentence repetition	0.05603	0.01912	2.930	0.00428 **

Signif. codes: 0 ‘***’ 0.001 ‘**’ 0.01 ‘*’ 0.05 ‘.’ 0.1 ‘ ’ 1

Autistic groups:

	Estimate	Std. Error	t value	Pr(>|t|)
(Intercept)	0.48448	3.48859	0.139	0.88986
bilinguals	4.25997	0.89202	4.776	6.91e-06 ***
age	0.09627	0.19115	0.504	0.61574
SES	-0.17169	0.10382	-1.654	0.10167
ADI-R total	0.00802	0.06822	0.118	0.90668
IQ	0.02689	0.01758	1.530	0.12949

loc-to-glo	0.05198	0.01594	3.261	0.00157 **
glo-to-loc	0.05345	0.01727	3.096	0.00262 **
expressive vocabulary	-0.06141	0.04745	-1.294	0.19883
sentence repetition	0.03063	0.01856	1.650	0.10240

Signif. codes: 0 ‘***’ 0.001 ‘**’ 0.01 ‘*’ 0.05 ‘.’ 0.1 ‘ ’ 1

Linear model results for syntactic interference word recall scores

TD groups:

	Estimate	Std. Error	t value	Pr(>|t|)
(Intercept)	13.040645	1.789006	7.289	1.1e-10 ***
bilinguals	0.794668	0.440283	1.805	0.0744 .
age	-0.562773	0.256589	-2.193	0.0308 *
SES	-0.033466	0.075979	-0.440	0.6606
loc-to-glo	0.006642	0.018555	0.358	0.7212
glo-to-loc	0.008152	0.007347	1.110	0.2701
expressive vocabulary	0.077212	0.041688	1.852	0.0672 .
sentence repetition	0.024620	0.011613	2.120	0.0367 *

Signif. codes: 0 '***' 0.001 '**' 0.01 '*' 0.05 '.' 0.1 ' ' 1

Autistic groups:

	Estimate	Std. Error	t value	Pr(>|t|)
(Intercept)	2.40511	4.14525	0.580	0.56322
bilinguals	2.72302	1.05992	2.569	0.01184 *
age	-0.22669	0.22714	-0.998	0.32093
SES	0.01937	0.12336	0.157	0.87558
ADI-R total	0.07755	0.08106	0.957	0.34131
IQ	-0.01319	0.02089	-0.632	0.52920
loc-to-glo	-0.01651	0.01894	-0.872	0.38559
glo-to-loc	-0.02776	0.02052	-1.353	0.17944

expressive vocabulary	0.16230	0.05638	2.879	0.00499 **
sentence repetition	0.02354	0.02206	1.067	0.28861

Signif. codes: 0 '***' 0.001 '**' 0.01 '*' 0.05 '.' 0.1 ' ' 1

Linear model results for sentence comprehension in the syntactic interference word recall task

TD groups:

	Estimate	Std. Error	t value	Pr(>|t|)
(Intercept)	12.347462	2.417101	5.108	1.78e-06 ***
bilinguals	-1.500822	0.594860	-2.523	0.0134 *
age	-0.348249	0.346674	-1.005	0.3178
SES	0.074509	0.102654	0.726	0.4698
loc-to-glo	-0.014792	0.025069	-0.590	0.5566
glo-to-loc	-0.004822	0.009926	-0.486	0.6283
expressive vocabulary	0.068186	0.056324	1.211	0.2292
sentence repetition	-0.002034	0.015690	-0.130	0.8972

Signif. codes: 0 ‘***’ 0.001 ‘**’ 0.01 ‘*’ 0.05 ‘.’ 0.1 ‘ ’ 1

Autistic groups:

	Estimate	Std. Error	t value	Pr(>|t|)
(Intercept)	3.808159	2.944577	1.293	0.199224
bilinguals	1.840296	0.752916	2.444	0.016466 *
age	-0.417980	0.161346	-2.591	0.011176 *
SES	-0.009187	0.087632	-0.105	0.916738
ADI-R total	0.010355	0.057584	0.180	0.857697
IQ	-0.002949	0.014836	-0.199	0.842875
loc-to-glo	-0.001332	0.013454	-0.099	0.921333

glo-to-loc	-0.056398	0.014573	-3.870	0.000206 ***
expressive vocabulary	0.174075	0.040047	4.347	3.63e-05 ***
sentence repetition	0.058146	0.015668	3.711	0.000357 ***

Signif. codes: 0 ‘***’ 0.001 ‘**’ 0.01 ‘*’ 0.05 ‘.’ 0.1 ‘ ’ 1

Linear model results for proactive interference list 2

TD groups:

	Estimate	Std. Error	t value	Pr(>|t|)
(Intercept)	-0.439709	1.176095	-0.374	0.7094
bilinguals	0.157715	0.289443	0.545	0.5872
age	-0.186903	0.168682	-1.108	0.2708
SES	-0.052193	0.049949	-1.045	0.2988
loc-to-glo	0.002790	0.012198	0.229	0.8196
glo-to-loc	-0.002861	0.004830	-0.592	0.5550
expressive vocabulary	0.061412	0.027406	2.241	0.0275 *
sentence repetition	-0.011157	0.007635	-1.461	0.1474

Signif. codes: 0 '***' 0.001 '**' 0.01 '*' 0.05 '.' 0.1 ' ' 1

Autistic groups:

	Estimate	Std. Error	t value	Pr(>|t|)
(Intercept)	-4.2202205	1.7148802	-2.461	0.0158 *
bilinguals	1.1172584	0.4384875	2.548	0.0125 *
age	-0.0777305	0.0939656	-0.827	0.4103
SES	0.0491788	0.0510354	0.964	0.3378
ADI-R total	0.0345292	0.0335362	1.030	0.3060
IQ	0.0160719	0.0086403	1.860	0.0661
loc-to-glo	-0.0092072	0.0078353	-1.175	0.2431
glo-to-loc	0.0167477	0.0084872	1.973	0.0515 .

expressive vocabulary	0.0001902	0.0233226	0.008	0.9935
sentence repetition	-0.0040782	0.0091246	-0.447	0.6560

Signif. codes: 0 '***' 0.001 '**' 0.01 '*' 0.05 '.' 0.1 ' ' 1

Linear model results for proactive interference list 3

TD groups:

<table>
<tr><th></th><th>Estimate</th><th>Std. Error</th><th>t value</th><th>Pr(>|t|)</th></tr>
<tr><td>(Intercept)</td><td>-2.652529</td><td>1.205375</td><td>-2.201</td><td>0.0303 *</td></tr>
<tr><td>bilinguals</td><td>0.216986</td><td>0.296649</td><td>0.731</td><td>0.4664</td></tr>
<tr><td>age</td><td>0.038777</td><td>0.172882</td><td>0.224</td><td>0.8230</td></tr>
<tr><td>SES</td><td>-0.008056</td><td>0.051192</td><td>-0.157</td><td>0.8753</td></tr>
<tr><td>loc-to-glo</td><td>-0.002464</td><td>0.012502</td><td>-0.197</td><td>0.8442</td></tr>
<tr><td>glo-to-loc</td><td>-0.003285</td><td>0.004950</td><td>-0.664</td><td>0.5087</td></tr>
<tr><td>expressive vocabulary</td><td>0.042811</td><td>0.028088</td><td>1.524</td><td>0.1309</td></tr>
<tr><td>sentence repetition</td><td>-0.009646</td><td>0.007825</td><td>-1.233</td><td>0.2208</td></tr>
</table>

Signif. codes: 0 '***' 0.001 '**' 0.01 '*' 0.05 '.' 0.1 ' ' 1

Autistic groups:

<table>
<tr><th></th><th>Estimate</th><th>Std. Error</th><th>t value</th><th>Pr(>|t|)</th></tr>
<tr><td>(Intercept)</td><td>-3.510409</td><td>1.975780</td><td>-1.777</td><td>0.0790 .</td></tr>
<tr><td>bilinguals</td><td>0.658751</td><td>0.505198</td><td>1.304</td><td>0.1956</td></tr>
<tr><td>age</td><td>0.155336</td><td>0.108261</td><td>1.435</td><td>0.1548</td></tr>
<tr><td>SES</td><td>-0.038620</td><td>0.058800</td><td>-0.657</td><td>0.5130</td></tr>
<tr><td>ADI-R total</td><td>-0.022001</td><td>0.038638</td><td>-0.569</td><td>0.5705</td></tr>
<tr><td>IQ</td><td>0.012017</td><td>0.009955</td><td>1.207</td><td>0.2305</td></tr>
<tr><td>loc-to-glo</td><td>-0.016872</td><td>0.009027</td><td>-1.869</td><td>0.0649 .</td></tr>
<tr><td>glo-to-loc</td><td>0.024778</td><td>0.009778</td><td>2.534</td><td>0.0130 *</td></tr>
</table>

expressive vocabulary	-0.015085	0.026871	-0.561	0.5759
sentence repetition	0.005719	0.010513	0.544	0.5878

Signif. codes: 0 '***' 0.001 '**' 0.01 '*' 0.05 '.' 0.1 ' ' 1

Autism effects

Linear model results for listening span scores

Monolingual groups:

	Estimate	Std. Error	t value	Pr(>|t|)
(Intercept)	3.108826	2.223454	1.398	0.16542
Autism	-2.389098	0.796038	-3.001	0.00346 **
age	-0.066664	0.210839	-0.316	0.75258
SES	0.147513	0.105352	1.400	0.16482
loc-to-glo	0.026286	0.013062	2.012	0.04710 *
glo-to-loc	-0.016520	0.009648	-1.712	0.09019 .
expressive vocabulary	0.015037	0.042816	0.351	0.72624
sentence repetition	0.031356	0.016000	1.960	0.05305 .

Signif. codes: 0 ‘***’ 0.001 ‘**’ 0.01 ‘*’ 0.05 ‘.’ 0.1 ‘ ’ 1

Bilingual groups:

	Estimate	Std. Error	t value	Pr(>|t|)
(Intercept)	3.93040	2.90545	1.353	0.17960
Autism	0.39933	0.67111	0.595	0.55335
age	0.27664	0.32239	0.858	0.39317
SES	-0.18729	0.11982	-1.563	0.12162
home language history	0.00436	0.01980	0.220	0.82627
current language use	0.02630	0.02850	0.923	0.35873

loc-to-glo	0.07793	0.04209	1.852	0.06743 .
glo-to-loc	0.01727	0.02434	0.709	0.48002
expressive vocabulary	-0.08024	0.07242	-1.108	0.27085
sentence repetition	0.05389	0.02022	2.665	0.00916 **

Signif. codes: 0 ‘***’ 0.001 ‘**’ 0.01 ‘*’ 0.05 ‘.’ 0.1 ‘ ’ 1

Linear model results for syntactic interference word recall scores

Monolingual groups:

	Estimate	Std. Error	t value	Pr(>|t|)
(Intercept)	10.079798	2.098961	4.802	6.06e-06 ***
Autism	-3.322128	0.751467	-4.421	2.69e-05 ***
age	-0.075568	0.199034	-0.380	0.70506
SES	-0.270150	0.099453	-2.716	0.00788 **
loc-to-glo	0.001037	0.012331	0.084	0.93319
glo-to-loc	0.006055	0.009107	0.665	0.50780
expressive vocabulary	0.058813	0.040419	1.455	0.14905
sentence repetition	0.045795	0.015104	3.032	0.00316 **

Signif. codes: 0 '***' 0.001 '**' 0.01 '*' 0.05 '.' 0.1 ' ' 1

Bilingual groups:

	Estimate	Std. Error	t value	Pr(>|t|)
(Intercept)	8.9958086	2.5135172	3.579	0.000564 ***
Autism	-1.6550794	0.5805776	-2.851	0.005433 **
age	-0.4048052	0.2789010	-1.451	0.150216
SES	0.1860760	0.1036573	1.795	0.076070
home language history	0.0070850	0.0171332	0.414	0.680229
current language use	0.0082400	0.0246571	0.334	0.739036
loc-to-glo	-0.0531194	0.0364102	-1.459	0.148148
glo-to-loc	0.0013045	0.0210593	0.062	0.950748

expressive vocabulary	0.1533059	0.0626480	2.447	0.016388 *
sentence repetition	0.0001031	0.0174967	0.006	0.995310

Signif. codes: 0 '***' 0.001 '**' 0.01 '*' 0.05 '.' 0.1 ' ' 1

Linear model results for sentence comprehension in the syntactic interference word recall task

Monolingual groups:

	Estimate	Std. Error	t value	Pr(>|t|)
(Intercept)	12.134666	2.136105	5.681	1.56e-07 ***
Autism	-3.281655	0.764765	-4.291	4.39e-05 ***
age	-0.513194	0.202557	-2.534	0.01298 *
SES	-0.045078	0.101213	-0.445	0.65709
loc-to-glo	0.014533	0.012549	1.158	0.24981
glo-to-loc	-0.011354	0.009269	-1.225	0.22369
expressive vocabulary	0.068202	0.041134	1.658	0.10072
sentence repetition	0.044227	0.015371	2.877	0.00499 **

Signif. codes: 0 '***' 0.001 '**' 0.01 '*' 0.05 '.' 0.1 ' ' 1

Bilingual groups:

	Estimate	Std. Error	t value	Pr(>|t|)
(Intercept)	6.689996	2.308284	2.898	0.00474 **
Autism	-0.379886	0.533172	-0.713	0.47804
age	-0.480033	0.256128	-1.874	0.06422
SES	0.084612	0.095193	0.889	0.37651
home language history	0.008296	0.015734	0.527	0.59936
current language use	0.009872	0.022644	0.436	0.66393
loc-to-glo	-0.014330	0.033437	-0.429	0.66928
glo-to-loc	-0.024976	0.019340	-1.291	0.19993

expressive vocabulary	0.189069	0.057533	3.286	0.00146 **
sentence repetition	0.010143	0.016068	0.631	0.52953

Signif. codes: 0 ‘***’ 0.001 ‘**’ 0.01 ‘*’ 0.05 ‘.’ 0.1 ‘ ’ 1

Linear model results for proactive interference list 2

Monolingual groups:

	Estimate	Std. Error	t value	Pr(>|t|)
(Intercept)	-0.5724066	1.1294394	-0.507	0.6135
Autism	-0.7816080	0.4043604	-1.933	0.0563
age	-0.0577123	0.1070993	-0.539	0.5913
SES	0.0382177	0.0535150	0.714	0.4769
loc-to-glo	-0.0092814	0.0066351	-1.399	0.1652
glo-to-loc	-0.0005149	0.0049007	-0.105	0.9166
expressive vocabulary	0.0075726	0.0217492	0.348	0.7285
sentence repetition	-0.0094432	0.0081275	-1.162	0.2483

Signif. codes: 0 '***' 0.001 '**' 0.01 '*' 0.05 '.' 0.1 ' ' 1

Bilingual groups:

	Estimate	Std. Error	t value	Pr(>|t|)
(Intercept)	-0.982192	1.187311	-0.827	0.4103
Autism	0.571664	0.274248	2.084	0.0400 * [1]
age	-0.191097	0.131744	-1.451	0.1505
SES	-0.022463	0.048965	-0.459	0.6475
home language history	0.009246	0.008093	1.142	0.2563
current language use	-0.008949	0.011647	-0.768	0.4443
loc-to-glo	0.040511	0.017199	2.355	0.0207 *
glo-to-loc	-0.006064	0.009948	-0.610	0.5437

expressive vocabulary	0.066377	0.029593	2.243	0.0274 *
sentence repetition	-0.010417	0.008265	-1.260	0.2109

Signif. codes: 0 '***' 0.001 '**' 0.01 '*' 0.05 '.' 0.1 ' ' 1

[1] Note: This effect does not reach significance after corrections for multiple comparisons

Linear model results for proactive interference list 3

Monolingual groups:

	Estimate	Std. Error	t value	Pr(>|t|)
(Intercept)	-1.8376597	1.1003803	-1.670	0.09831
Autism	-0.6535959	0.3939567	-1.659	0.10051
age	0.0440049	0.1043438	0.422	0.67421
SES	0.0607148	0.0521382	1.164	0.24723
loc-to-glo	-0.0190807	0.0064644	-2.952	0.00401 **
glo-to-loc	-0.0004711	0.0047746	-0.099	0.92161
expressive vocabulary	-0.0032202	0.0211896	-0.152	0.87954
sentence repetition	-0.0054781	0.0079184	-0.692	0.49079

Signif. codes: 0 '***' 0.001 '**' 0.01 '*' 0.05 '.' 0.1 ' ' 1

Bilingual groups:

	Estimate	Std. Error	t value	Pr(>|t|)
(Intercept)	-2.8444108	1.3866974	-2.051	0.0432 *
Autism	0.2456045	0.3203024	0.767	0.4453
age	0.0724477	0.1538685	0.471	0.6389
SES	-0.0963413	0.0571873	-1.685	0.0956
home language history	0.0047937	0.0094523	0.507	0.6133
current language use	-0.0016641	0.0136032	-0.122	0.9029
loc-to-glo	0.0402536	0.0200873	2.004	0.0482 *

glo-to-loc	-0.0008398	0.0116183	-0.072	0.9425
expressive vocabulary	0.0467692	0.0345627	1.353	0.1795
sentence repetition	-0.0050227	0.0096528	-0.520	0.6041

Signif. codes: 0 '***' 0.001 '**' 0.01 '*' 0.05 '.' 0.1 ' ' 1
